# MRI-based pre-Radiomics and delta-Radiomics models accurately predict the post-treatment response of rectal adenocarcinoma to neoadjuvant chemoradiotherapy

**DOI:** 10.3389/fonc.2023.1133008

**Published:** 2023-02-22

**Authors:** Likun Wang, Xueliang Wu, Ruoxi Tian, Hongqing Ma, Zekun Jiang, Weixin Zhao, Guoqing Cui, Meng Li, Qinsheng Hu, Xiangyang Yu, Wengui Xu

**Affiliations:** ^1^ Tianjin’s Clinical Research Center for Cancer, Tianjin Medical University Cancer Institute and Hospital, Tianjin, China; ^2^ Department of Molecular Imaging and Nuclear Medicine, National Clinical Research Center for Cancer, Key Laboratory of Cancer Prevention and Therapy, Tianjin, China; ^3^ Department of Ultrasound Medicine, The First Affiliated Hospital of Hebei North University, Zhangjiakou, China; ^4^ Graduate School, Tianjin Medical University, Tianjin, China; ^5^ Department of Gastrointestinal Surgery, Tianjin Medical University Nankai Hospital, Tianjin, China; ^6^ Department of Colorectal Surgery, National Cancer Center, National Clinical Research Center for Cancer, Cancer Hospital, Chinese Academy of Medical Sciences, Beijing, China; ^7^ Department of General Surgery, The Fourth Hospital of Hebei Medical University, Shijiazhuang, China; ^8^ College of Computer Science, Sichuan University, Chengdu, China; ^9^ Medical Image Center, The First Affiliated Hospital of Hebei North University, Zhangjiakou, China; ^10^ Graduate School, Hebei North University, Zhangjiakou, China; ^11^ Department of Orthopedics, West China Hospital, Sichuan University, Chengdu, Sichuan, China

**Keywords:** rectal adenocarcinoma, neoadjuvant chemoradiotherapy, MRI, radiomics, machine learning

## Abstract

**Objectives:**

To develop and validate magnetic resonance imaging (MRI)-based pre-Radiomics and delta-Radiomics models for predicting the treatment response of local advanced rectal cancer (LARC) to neoadjuvant chemoradiotherapy (NCRT).

**Methods:**

Between October 2017 and August 2022, 105 LARC NCRT-naïve patients were enrolled in this study. After careful evaluation, data for 84 patients that met the inclusion criteria were used to develop and validate the NCRT response models. All patients received NCRT, and the post-treatment response was evaluated by pathological assessment. We manual segmented the volume of tumors and 105 radiomics features were extracted from three-dimensional MRIs. Then, the eXtreme Gradient Boosting algorithm was implemented for evaluating and incorporating important tumor features. The predictive performance of MRI sequences and Synthetic Minority Oversampling Technique (SMOTE) for NCRT response were compared. Finally, the optimal pre-Radiomics and delta-Radiomics models were established respectively. The predictive performance of the radionics model was confirmed using 5-fold cross-validation, 10-fold cross-validation, leave-one-out validation, and independent validation. The predictive accuracy of the model was based on the area under the receiver operator characteristic (ROC) curve (AUC).

**Results:**

There was no significant difference in clinical factors between patients with good and poor reactions. Integrating different MRI modes and the SMOTE method improved the performance of the radiomics model. The pre-Radiomics model (train AUC: 0.93 ± 0.06; test AUC: 0.79) and delta-Radiomcis model (train AUC: 0.96 ± 0.03; test AUC: 0.83) all have high NCRT response prediction performance by LARC. Overall, the delta-Radiomics model was superior to the pre-Radiomics model.

**Conclusion:**

MRI-based pre-Radiomics model and delta-Radiomics model all have good potential to predict the post-treatment response of LARC to NCRT. Delta-Radiomics analysis has a huge potential for clinical application in facilitating the provision of personalized therapy.

## 1 Introduction

Locally advanced middle-low rectal cancer (LARC) refers to the rectal tumor less than or equal to 10 cm away from the rectal margin, which is at stages T3 or T4 or N+, and M0 (1). Because of the small space between the rectal and pelvic structures and organs, the absence of serous membrane in the rectum, and the difficulty in obtaining sufficient circumferential margin (CRM+) during surgery, LARC has a very high local recurrence rate, low anal preservation rate, and higher chances of complications and poor quality of life of patients (2, 3). Therefore, neoadjuvant therapy (NCRT), including the preoperative chemoradiotherapy, total mesorectum excision (TME) plus postoperative adjuvant therapy (sandwich model), and neoadjuvant therapy plus TME (TNT model) have recently been recommended in the latest edition of the National Comprehensive Cancer Network (NCCN) guidelines and the 2020 Chinese colorectal Cancer Diagnosis and Treatment guidelines to treat LARC (1, 4). Compared with the surgery plus postoperative adjuvant chemotherapy, NCRT significantly reduces the local recurrence rate, increases the R0 resection rate, and prolongs the survival of patients with LARC. In addition, NCRT has a better local control rate and is only associated with fewer adverse reactions than traditional postoperative adjuvant therapy (5).

The pathological complete response rate (pCR) of preoperative neoadjuvant therapy for patients with LARC is about 20% (6–9). On the other hand, some studies have shown that the pCR of NCRT combined with immunotherapy could be higher than 40% and the rate of patients with apparent/moderate retreatment could be between 20%-30%, so NCRT has significant downstaging effect. However, NCRT may also lead to severe adverse reactions, such as fecal incontinence, gastric emptying disorder, radiation enteritis, sexual dysfunction, bone marrow suppression, gastrointestinal side reactions, and neurotoxicity. In addition, a small proportion of patients do not respond to the treatment (non-sensitive to radiation and chemotherapy/immune therapy). Therefore, it is crucial to accurately evaluate the effect of neoadjuvant therapy before surgery and develop individualized therapy, mainly for patients who are sensitive to the therapy, while patients with intolerant and nonresponse to neoadjuvant therapy could be treated with other therapies and surgery in order to effectively avoid the toxicity of chemoradiotherapy, which is the focus of current neoadjuvant therapy for LARC (10, 11).

Radiomics analysis, which extracts a large number of mineable features from medical images using data characterization algorithms, has the potential to uncover disease characteristics that are difficult to identify by human vision alone (12, 13). In the last two years, several studies have shifted the attention towards constructing novel radiomics models to predict the NCRT response of LARC. Most studies have already demonstrated the application of radiomics features based on pre-therapy MRI for predicting the treatment response of LARC after NCRT (14–28). Some studies are based only on T2 MRI (21, 23, 25, 26) or apparent diffusion coefficient (ADC) map (15), and their multi-modal imaging information is not validated. These studies (16, 19, 27) have found additional valuable perspectives using multi-modal MRI analysis. Furthermore, in some studies (29), tumor regression degree (mrTRG) on MRI was determined based on changes in tumor size and signal intensity on T1, T2, and WI to predict the outcome of NCRT. Unfortunately, as demonstrated, there is no pathological gold standard. The above studies only considered the contribution of pre-therapy images and did not include a comprehensive analysis of images before and after the therapy. Only a few studies have focused on the radiomics models at different time nodes (30, 31).

This study aimed to develop and validate the novel models for predicting LARC response to NCRT based on machine learning algorithms using radiomics features (T1, T2, and T1+T2) obtained from pre-therapy and post-therapy MRI images of LARC patients.

## 2 Materials and methods

The protocol for this retrospective study was approved by the ethics committee of The First Affiliated Hospital of Hebei North University and The Fourth Hospital of Hebei Medical University. Patient approval or informed consent for the review of medical images was not necessary.

### 2.1 Patients

**Figure 1:**
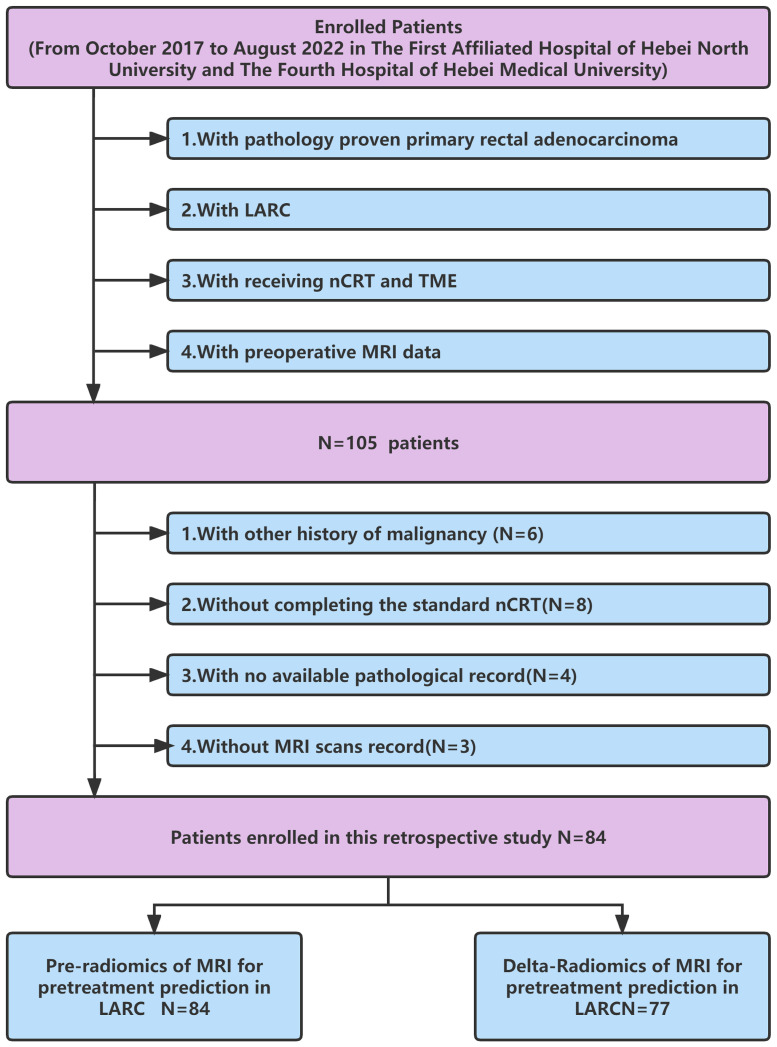
Flow chart for inclusion and exclusion criteria of the LARC patients. LARC, Local advanced rectal cancer; nCRT, neoadjuvant therapy; TME, total mesorectum excision.

The electronic medical database contained data for 105 rectal adenocarcinoma patients (adenocarcinoma < 10 cm from rectal lower margin) who underwent the standard long-course NCRT followed by radical resection between October 2017 and August 2022 at The First Affiliated Hospital of Hebei North University and The Fourth Hospital of Hebei Medical University. The inclusion criteria were as follows: (1) histologically diagnosed with primary rectal adenocarcinoma; (2) with locally advanced rectal cancer based on enhanced chest, abdominal, and pelvic CT, rectal MRI, and transrectal ultrasound, according to the eighth edition of the Joint Cancer Board (AJCC) (32) before treatment; (3) receiving neoadjuvant chemoradiotherapy and TME; (4) with preoperative MRI data. The exclusion criteria were as follows: (1) with incomplete standard NCRT. Eight patients did not complete the standard NCRT due to intolerance and rejection; (2) with other malignancies (six); (3) with no tumor regression grading data (four); (4) with low-quality key MRI images for analysis (three). In the end, 84 patients met the inclusion criteria. Further examination revealed that postoperative imaging data for 7 patients were missing. Finally, 84 patients with pre-Radiomics of MRI and 77 patients with delta-Radiomics MRI were included in this study (**Figure 1**).

According to the time of data collection, we used the last 16 data collected as an independent validation cohort, and the other data as the primary cohort for model construction and cross-validation. Finally, the established pre-Radiomics and delta-Radiomics models were evaluated again with the validation cohort.

### 2.2 Neoadjuvant chemoradiotherapy

Patients with LARC received long-course radiotherapy (45–50Gy, 1.8–2.0 Gy each time, 5 times per week) and concomitant chemotherapy (capecitabine 1250 mg/m2 twice a day, 5 times per week). Radical excision (TME) was performed within 8-12 weeks after the completion of NCRT.

### 2.3 Pathological assessment and tumor regression response

Postoperative TNM restaging was performed according to the pathological outcomes of the surgically resected specimens to evaluate the down-staging. Tumor regression response was evaluated systematically according to tumor regression grade (TRG) (32). The details were as follows: Grade 0: the tumor completely retracted, and only calcium salt deposition in the fibrous tissue showed pathological response; Grade 1,: moderate retraction. Here, fibrosis was present with a few visible tumor cells or cell masses; Grade 2: slight retraction. Here, there was no residual tumor, but strong fibrosis interstitial filling was present; Grade 3: no regression, extensive residual tumor, and little or no tumor cell necrosis. TRG0-1 was defined as a good reaction, whereas TRG2-3 was defined as a poor reaction.

### 2.4 MRI protocol

In this study, all MRI image data were acquired from two time points: one before NCRT and the other after NCRT. The pre-Radiomics study was conducted using pre-therapy MRI images, and the delta-Radiomics study was conducted using pre-therapy and post-therapy MRI images. All rectum MRI examinations were performed using a 3.0-T magnet (Philips Ingenia 3.0T) with a phased array surface coil. Bowel preparation was performed before image acquisition. The following pulse sequences covering the entire tumor were included: (1) axial (perpendicular to the long axis of the rectum) T2-weighted imaging (T2WI). This was obtained with a slice thickness of 3.8 mm, repetition time (TR)/echo time (TE) of 4000 ms/120 ms, a field of view (FOV) of 16 × 16 cm, matrix size of 320 × 256, echo train length (ETL) of 22, and the number of excitation (NEX) of 2.

### 2.5 Tumor segmentation

**Figure 2:**
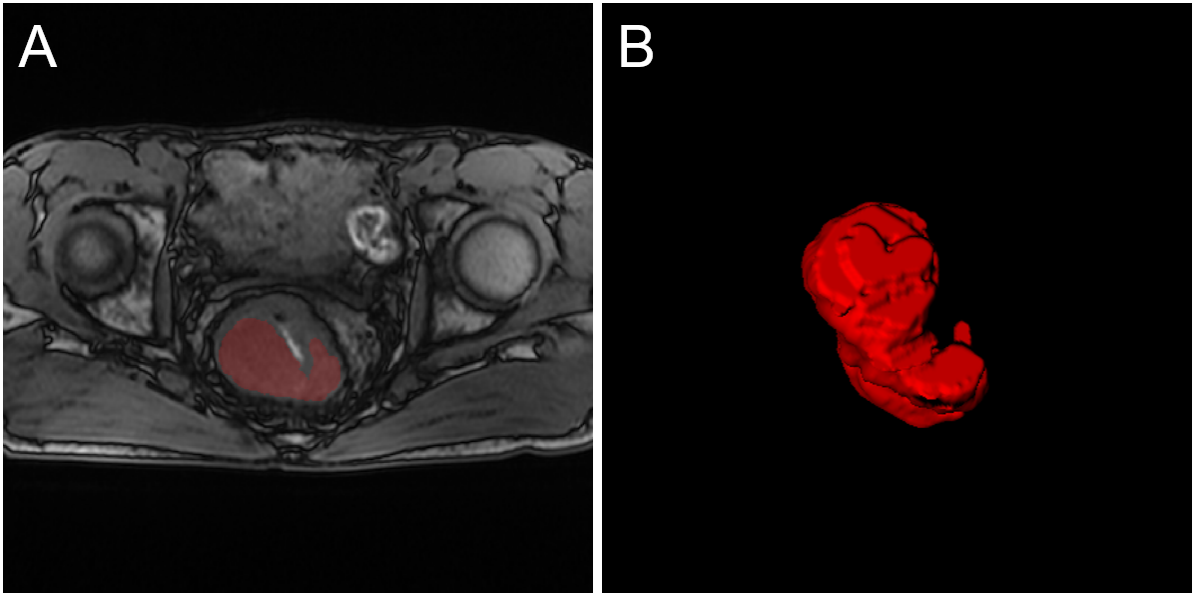
An example of tumor segmentation from T1 MRI. **(A)** The original image and tumor ROI; **(B)** The three-dimensional display of the entire tumor area.

First, T1 and T2 MRI images were normalized and aligned to facilitate accurate manual segmentation of tumor areas. Then, the regions of interest (ROIs) of the tumors were manually segmented using ITK-SNAP software by two experienced doctors (version 3.8.0; www.itksnap.org). Intraobserver difference of ROI was performed by calculating the Dice ratio. Segmentations with a dice ratio of over 0.90 were considered qualified. For those less than 0.90, the segmentation would be re-evaluated by a third experienced radiologist. An example of tumor segmentation is shown in **Figure 2**.

### 2.6 Pre-Radiomics and delta-Radiomics analysis

The image processing and radiomics feature extraction were performed using the Pyradiomics tool (version 3.0.1) as previously described (33, 34). A total of 105 three-dimensional Radiomics features from each tumor volume, including 14 shape-based features, 18 first-order features, and 73 texture features, were quantified. The three-dimensional texture features were calculated using gray level co-occurrence matrix (GLCM) (N=22), gray level size zone matrix (GLSZM) (N=14), gray level size zone matrix (GLSZM) (N=16), gray level run length matrix (GLRLM) (N=16), and neighboring gray-tone difference matrix (NGTDM) (N=5).

In the study, two radiomics types were defined: pre-Radiomics and delta-Radiomics. Pre-Radiomics analysis was implemented only using the pre-Radiomics features from pre-therapy MRI. Delta-Radiomics analysis was implemented using the delta-Radiomics features based on changes in radiomic features before and after NCRT, which were post-therapy radiomics features minus the pre-therapy radiomics features. All the imaging features were standardized for the subsequent machine-learning processing.

Then, eXtreme Gradient Boosting (XGBoost) was used to evaluate and select the important features, with the gbtree booster, a max-depth of 10, a lambda of 1, and an eta of 0.01, which was implemented using xgboost Python (version 0.82). Previous studies have also demonstrated that the XGBoost algorithm could be used for processing structured tabular data (35, 36). Based on experience, a feature in more than 10 samples or patients could be more robust for building binary classifiers (37, 38). Therefore, according to the sample size, an appropriate number of features were selected to construct the feature dataset. The sample imbalance was addressed using the Synthetic Minority Oversampling Technique (SMOTE) (39) to enhance the data and improve the modeling performance. Finally, the pre-Radiomics and delta-Radiomcis models were built using the corresponding features and XGBoost classifier.

### 2.7 Experimental details

Multiple group comparison experiments were performed. First, machine learning models were compared using single-model MRI and multi-modal MRI. The T1, T2, and T1+T2 integrated models were then constructed. Second, original features-based models and resampled features-based models using SMOTE were compared. Then, the pre-Radiomics model and delta-Radiomics model were compared through cross-validation with 5-fold and 10-fold, leave-one-out validation, and independent test. All the models were evaluated using the area under the receiver operator characteristic (ROC) curve (AUC). The degree of importance and statistical differences of valuable features and radiomics prediction scores in post-treatment responses were also assessed.

### 2.8 Statistical analysis

The relevant statistical analyses and machine learning algorithms were generated using Python (version 3.6.6). Differences between differently distributed variables were compared using T-test or Mann–Whitney U test. XGBoost was performed for feature selection and modeling. The prediction performance of the model was evaluated using the area under the ROC curves and mean AUCs through cross-validation. A Delong test was performed to compare the performance of the models. *P*-value < 0.05 was considered statistically significant.

## 3 Results

### 3.1 Clinical characteristics

Patient demographic characteristics are shown in **Table 1**. There was no significant difference in clinical factors between patients with good reactions and poor reactions to LARC. The reliability of results from small sample sizes is usually low (40, 41). Among the 84 study lesions for pre-Radiomics analysis, 28 (33.33%) were classified as having a good reaction, and 56 (66.67%) in the poor reaction group. For delta-Radiomcis analysis with 77 lesions, 27 (35.06%) were good reactions and 50 (64.94%) were poor reactions.

**Table 1:** Patient demographic characteristics.

Characteristics	Pre-Radiomics		Delta-Radiomics	
	Primary cohort (N=68)	Validation cohort (N=16)	Primary cohort (N=61)	Validation cohort (N=16)
Age				
≤60	30 (44.12%)	12 (75.00%)	28 (45.90%)	12 (75.00%)
>60	38 (55.88%)	4 (25.00%)	33 (54.10%)	4 (25.00%)
Gender, n (%)				
Male	50 (73.53%)	9 (56.25%)	45 (73.77%)	9 (56.25%)
Female	18 (26.47%)	7 (43.75%)	16 (26.23%)	7 (43.75%)
Tumor location cm				
Middle (5-10)	39 (57.35%)	9 (56.25%)	33 (54.10%)	9 (56.25%)
Low (≤5)	29 (42.65%)	7 (43.75%)	28 (45.90%)	7 (43.75%)
Tumor size, cm				
≤5	40 (58.82%)	10 (62.50%)	38 (62.30%)	10 (62.50%)
>5	28 (41.18%)	6 (37.50%)	23 (37.70%)	6 (37.50%)
Differentiated degree				
moderatel	53 (77.94%)	11 (68.75%)	48 (78.69%)	11 (68.75%)
Low	15 (22.06%)	5 (31.25%)	13 (21.31%)	5 (31.25%)
Serum CEA ng/ml				
>5.0	37 (54.41%)	4 (25.00%)	32 (52.46%)	4 (25.00%)
≤5.0	31 (45.59%)	12 (75.00%)	29 (47.54%)	12 (75.00%)
Clinical T stage, n (%)				
cT2-3	37 (54.41%)	6 (37.50%)	34 (55.74%)	6 (37.50%)
cT4	31 (45.59%)	10 (62.50%)	27 (44.26%)	10 (62.50%)
Clinical N stage, n (%)				
N0	6 (8.82%)	1 (6.25%)	6 (9.84%)	1 (6.25%)
N+	62 (91.18%)	15 (93.75%)	55 (90.16%)	15 (93.75%)
Response to NCRT, n (%)				
Good (0-1)	16 (23.53%)	12 (75.00%)	15 (24.59%)	12 (75.00%)
Poor (2-3)	52 (76.47%)	4 (25.00%)	46 (75.41%)	4 (25.00%)

### 3.2 Prediction performance across T1, T2, and T1+T2 models

**Figure 3:**
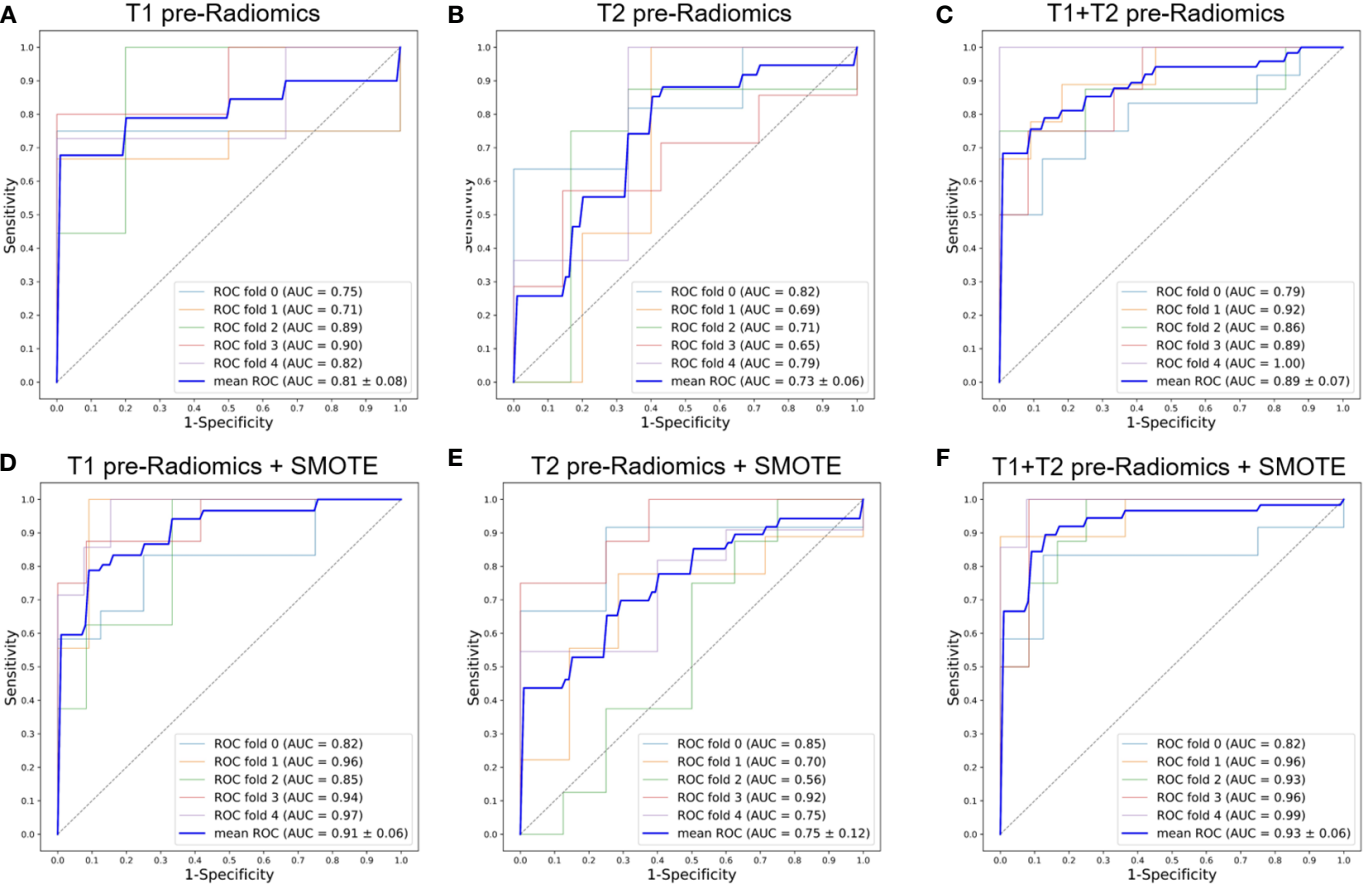
ROC curves of the pre-Radiomics models with 5-fold cross-validation. **(A)** T1 pre-Radiomics model; **(B)** T2 pre-Radiomics model; **(C)** T1+T2 pre-Radiomics model; **(D–F)** The models developed by incorporating the SMOTE method. ROC, receiver operator characteristic; AUC, area under the curve; SMOTE, Synthetic Minority Oversampling Technique.

**Figure 4:**
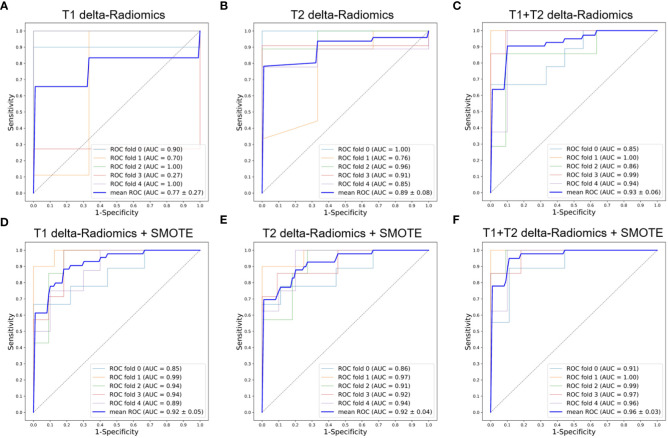
ROC curves of the delta-Radiomics models with 5-fold cross-validation. **(A)** T1 delta-Radiomics model; **(B)** T2 delta-Radiomics model; **(C)** T1+T2 delta-Radiomics model; **(D–F)** The above models were developed by incorporating the SMOTE method. ROC, receiver operator characteristic; AUC, area under the curve; SMOTE, Synthetic Minority Oversampling Technique.

**Figures 3A–C**, **4A–C** show the ROC curves for T1, T2, and T1+T2 models based on pre-Radiomcis and delta-Radiomcis analysis. In pre-Radiomics analysis, the mean AUC of the T1 model was 0.81, that of T2 was 0.73, and that of T1+T2 was 0.89. In delta-Radiomcis analysis, the mean AUC of T1 was 0.77, that of T2 was 0.89, and that of the T1+T2 model was 0.93. Therefore, T1 is more relevant than T2 in pre-Radiomics analysis, but T2 is more relevant in delta-Radiomcis. Based on the Delong test, combining the T1 and T2 models were superior to either model alone (P < 0.05).

### 3.3 Effect of SMOTE on modeling

**Figures 3**, **4** show the ROC curves of the original features-based models and SMOTE-based models. It was found that the oversampled data using SMOTE method is more accurate than original imbalanced data. As shown in **Figures 3D–F**, **4D–F** showed the same diagnostic trends and patterns as before, meaning that the SMOTE technology has superior data mining potential.

### 3.4 Valuable radiomics features

Based on the above experiments, we selected T1+T2 as the final radiomics model using SMOTE. The top-5 valuable pre-Radiomics and delta-Radiomics features are shown in **Table 2**. All the valuable pre-Radiomics features were from T1 MRI and texture features. T2 MRI had more significance in delta-Radiomics analysis than in pre-Radiomics. Overall, the delta-Radiomics features were more important than pre-Radiomcis features. The types of important pre-therapy imaging features were different from post-therapy features.

**Table 2:** The top-5 valuable pre-Radiomics and delta-Radiomics features.

Model	MRI sequences	Type	Features^*^	Importance^**^
pre-Radiomics	T1	GLDM	Small Dependence Low Gray Level Emphasis	406
	T1	GLSZM	Zone Entropy	374
	T1	GLCM	Idn	336
	T1	GLSZM	Size Zone Non-Uniformity Normalized	304
	T1	GLCM	Inverse Variance	284
delta-Radiomics	T1	Shape	Sphericity	492
	T2	GLCM	Cluster Prominence	338
	T2	Shape	Sphericity	304
	T2	GLDM	Dependence Non-Uniformity Normalized	270
	T1	First-order	90 Percentile	234

^*^ The mathematical definition of the radiomics features could be obtained at https://pyradiomics.readthedocs.io/en/latest/features.html.

^**^ The important coefficient was defined by XGBoost. The value is directly proportional to the degree of contribution to classifier modeling.

GLDM: gray level dependence matrix; GLSZM: gray-level size zone matrix; GLCM: gray-level co-occurrence matrix.

### 3.5 Comparison of the pre-Radiomics model and delta-Radiomics model

The final pre-Radiomics and delta-Radiomics models were built. The 5-fold cross-validation, 10-fold cross-validation, and leave-one-out validation were used to comprehensively evaluate the differences in the prediction performance between the two methods. The prediction performance of the pre-Radiomics and delta-Radiomics models is shown in **Table 3**. The predictive accuracy of the delta-Radiomcis model was higher than that of pre-Radiomcis model in 5-fold cross-validation (0.96 vs. 0.93), 10-fold cross-validation (0.95 vs. 0.92), and leave-one-out validation (0.93 vs. 0.90). The accuracy of all radiomics models was higher than 0.90, demonstrating their satisfactory good predictability in predicting the NCRT response of LARC.

**Table 3:** Prediction performance of the pre-Radiomics and delta-Radiomics models.

Model	mAUC^1*^	mAUC^2**^	mAUC^3***^	test AUC
pre-Radiomics	0.93 ± 0.06	0.92 ± 0.06	0.90 ± 0.07	0.79
delta-Radiomics	0.96 ± 0.03	0.95 ± 0.05	0.93 ± 0.06	0.83

^*^mAUC^1^ means the mean AUC based on 5-fold cross-validation.

^**^mAUC^2^ means the mean AUC based on 10-fold cross-validation.

^***^mAUC^3^ means the mean AUC based on the leave-one-out method.

**Figure 5:**
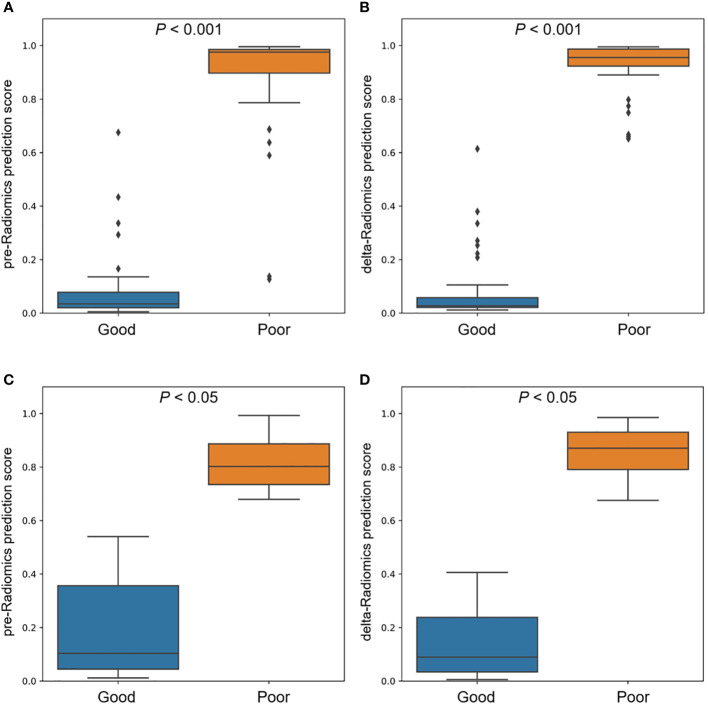
Comparison of pre-Radiomics and delta-Radiomics prediction scores between good and poor response groups. **(A)** pre-Radiomics prediction and **(B)** delta-Radiomics prediction in the primary cohort. **(C)** pre-Radiomics prediction and **(D)** delta-Radiomics prediction in the validation cohort.

A given cutoff prediction value for the models was selected to evaluate their NCRT prediction accuracy. The prediction probability of the machine learning models as the radiomics prediction scores to evaluate the degree of resistance to the treatment response. A higher score means a higher risk of poor reaction. **Figure 5** shows the prediction scores of the pre-Radiomics and delta-Radiomics between good and poor response groups in the primary and validation cohort. Both models accurately distinguished responses to NCRT in the primary cohort (P < 0.001) and the validation cohort (P < 0.05).

## 4 Discussion

In this study, we developed and validated the novel MRI-based pre-Radiomics and delta-Radiomics models to predict the treatment response of LARC to NCRT. The results showed that the predictive accuracy of these models was very high and robust, and delta-Radiomics could be used as an imaging biomarker for clinical transformation.

Studies have shown that radiomics models based on preoperative T1 and T2 and delta-Radiomics have a good predictive performance of LARC to NCRT (25–27, 30, 31), consistent with our findings. We also found that to some extent, integrating the multi-modal imaging data improve the predictive performance of the radiomics models, and sample balancing with the SMOTE technique can uncover the pattern of radiomics data.

In addition to building machine learning models, we also found that the texture features of the images contributes to the prediction of NCRT response by LARC, consistent with previous studies (26, 28). Moreover, wavelet transformation may enhance the texture characteristics of the images, improving the model performance (42), which may give some hints that this task can be verified in future studies.

In addition to the MRI-based radiomics research, other deep learning models have achieved remarkable results (43, 44). Many other machine learning tools built from other data modalities to predict LARC response to NCRT have also been developed (45–48). Medical multi-modal information fusion is an inevitable development trend in intelligent precision medicine. Multi-modal data could be used to build more efficient and robust clinical diagnostic tools through extensive reference to other successful studies.

This research has potential for future improvement. First, the sample size was small, which limited the upper limit of data mining and model building. Although data was obtained from two centers and adopted a data enhancement algorithm, there is still some bias. Second, several cross-validation algorithms were used to evaluate the overall performance of our model. Through 5-fold cross-validation, 10-fold cross-validation, leave-one-out validation, and independent validation, the good predictive performance of the radiomics models was confirmed, which explains the generalization of the models to a certain extent. But the validation of large scale multi-center data cohort is the best way to evaluate and transform imaging biomarkers. Finally, because this was a retrospective study, we had no control over the collected data. Thus, key additional clinical data that could have enhanced our research outcome could not be included. Future studies should consider incorporating multi-modal data to build a better predictive model.

## 5 Conclusion

This study demonstrated that MRI-based pre-Radiomics and delta-Radiomics models could accurately predict the post-treatment response of LARC to NCRT. Delta-Radiomcis analysis may also be used in the clinical diagnosis of LARC for personalized medicine.

## Data availability statement

The original contributions presented in the study are included in the article/supplementary material. Further inquiries can be directed to the corresponding authors.

## Ethics statement

The studies involving human participants were reviewed and approved by the ethic committee of The First Affiliated Hospital of Hebei North University and The Fourth Hospital of Hebei Medical University. Written informed consent for participation was not required for this study in accordance with the national legislation and the institutional requirements.

## Author contributions

LW, XW, and ZJ: study design. XW, RT, HM, ML, XY, and WX: data collection. ZJ and WZ: data analysis. QH, XY, and WX: supervision. LW, XW, and ZJ: manuscript writing. All authors contributed to the article and approved the submitted version.
